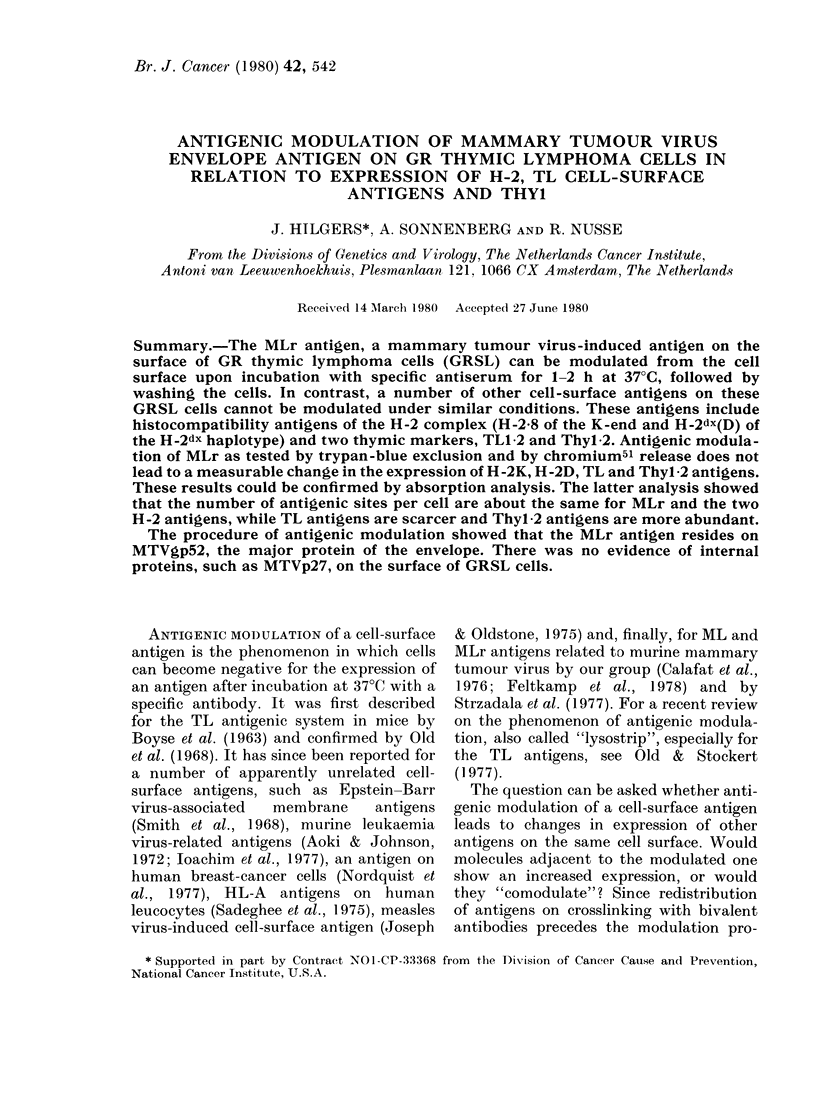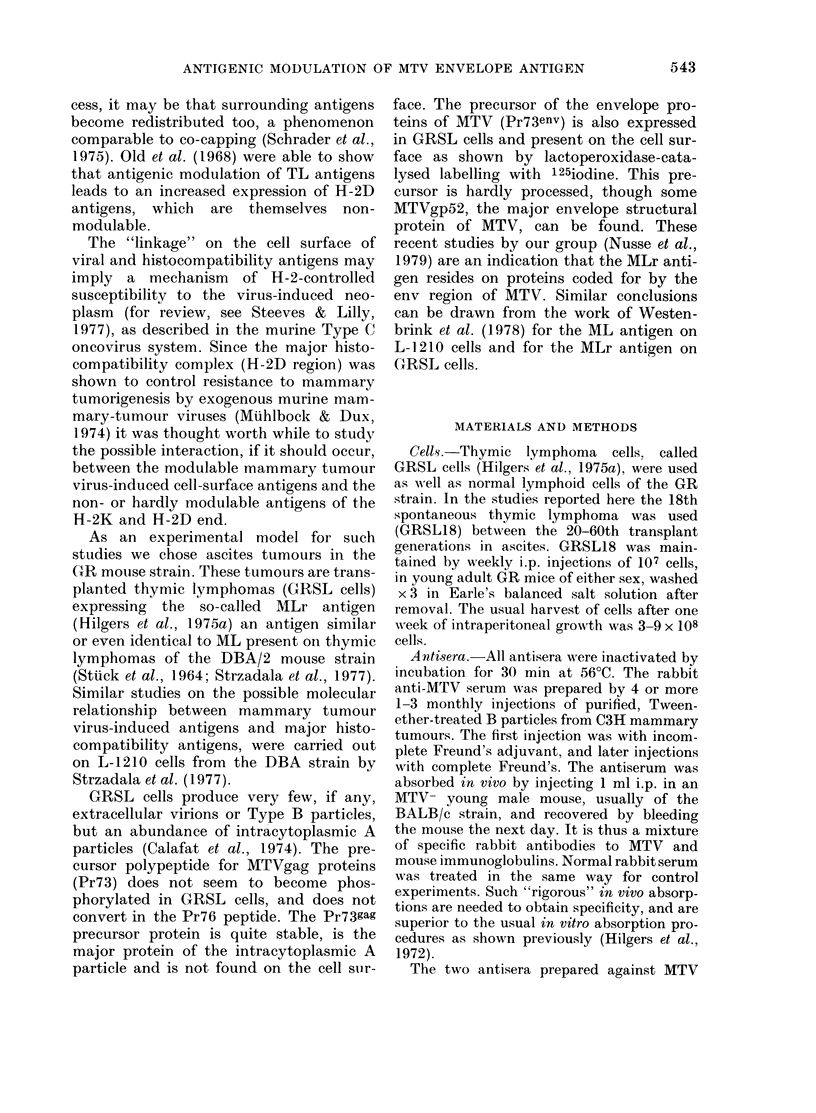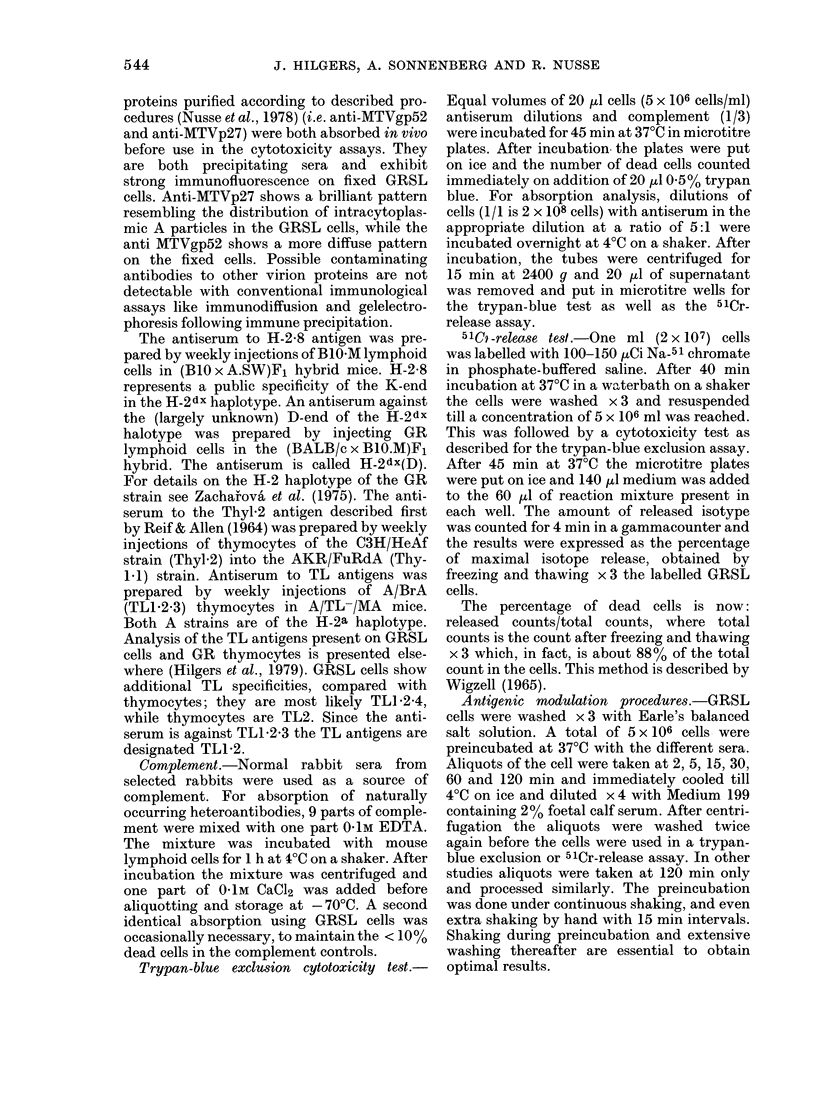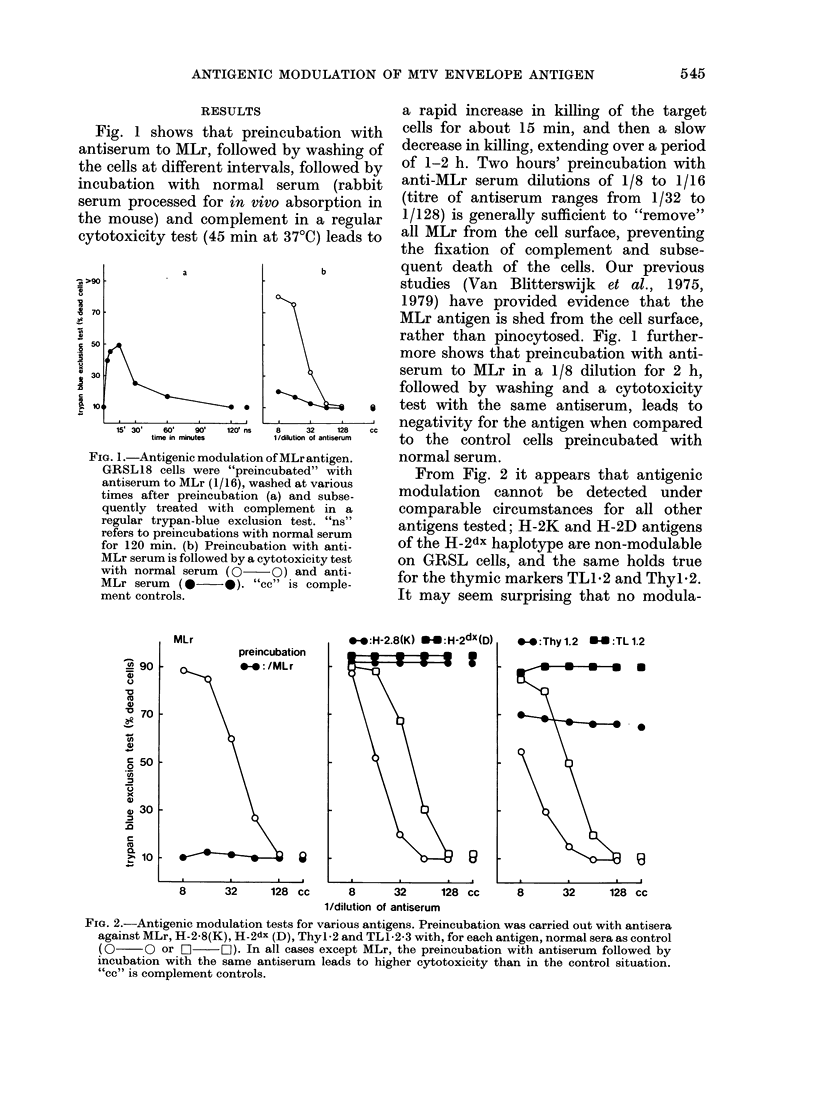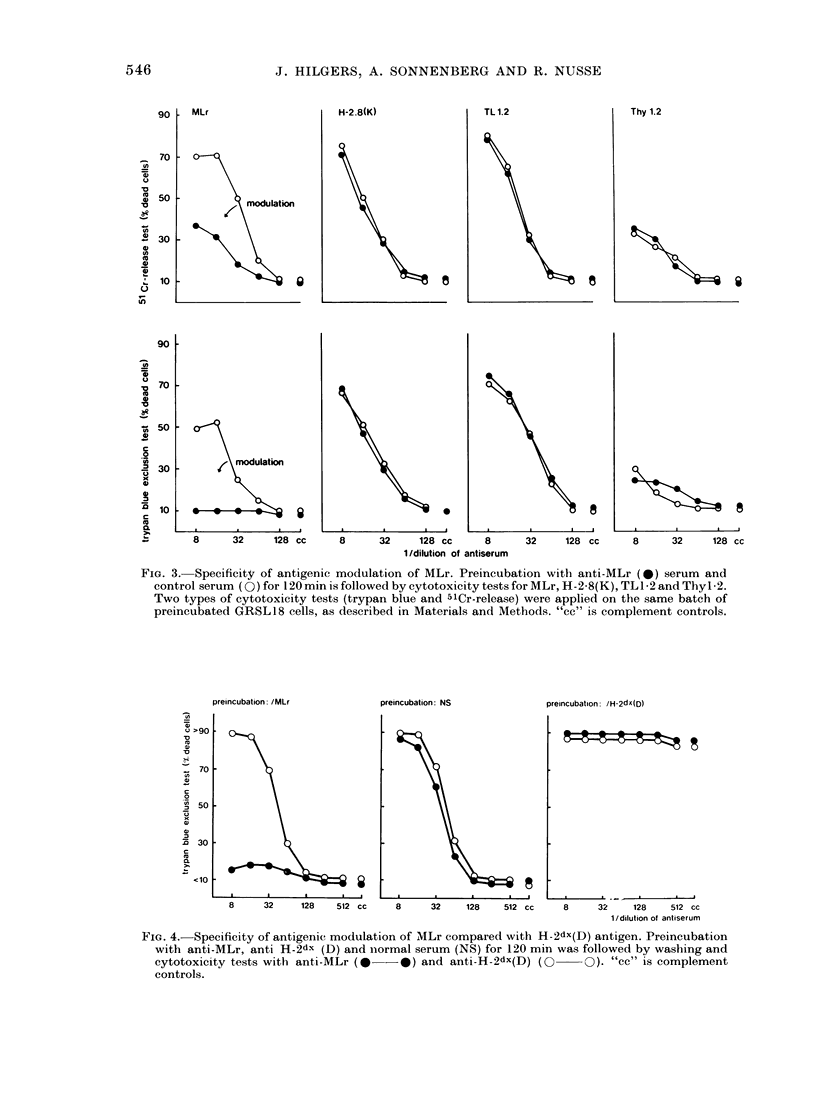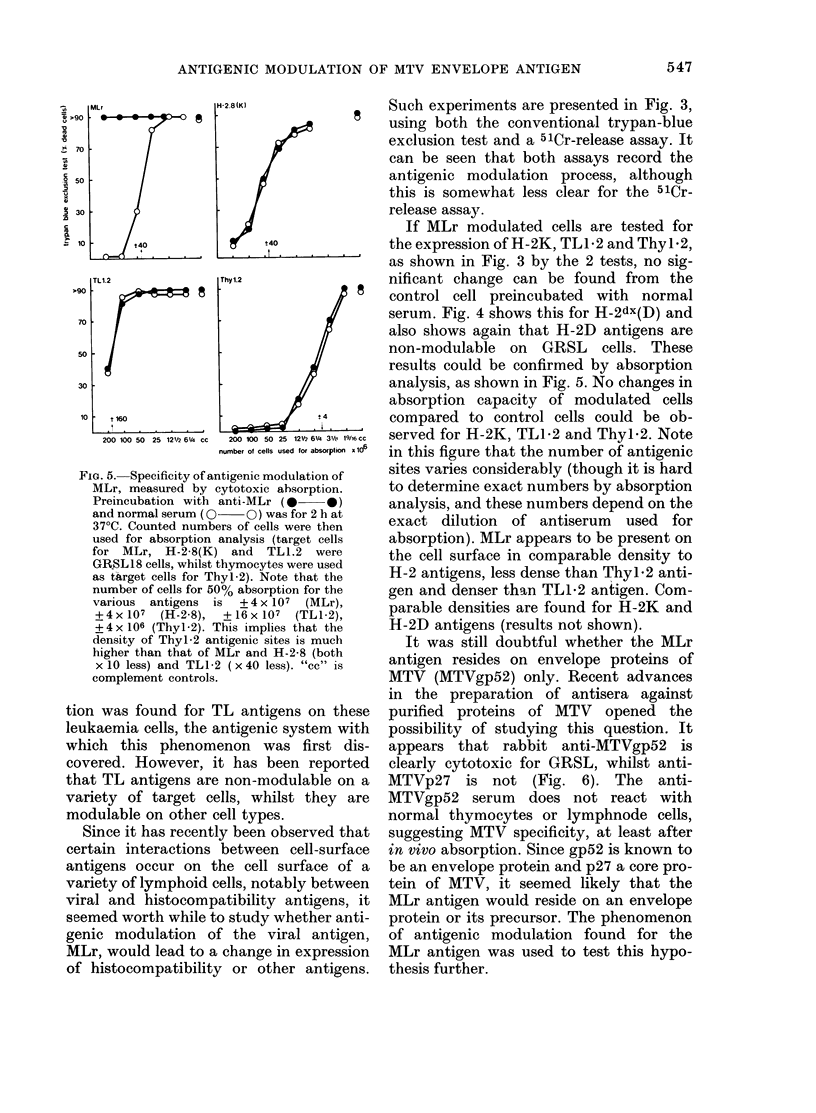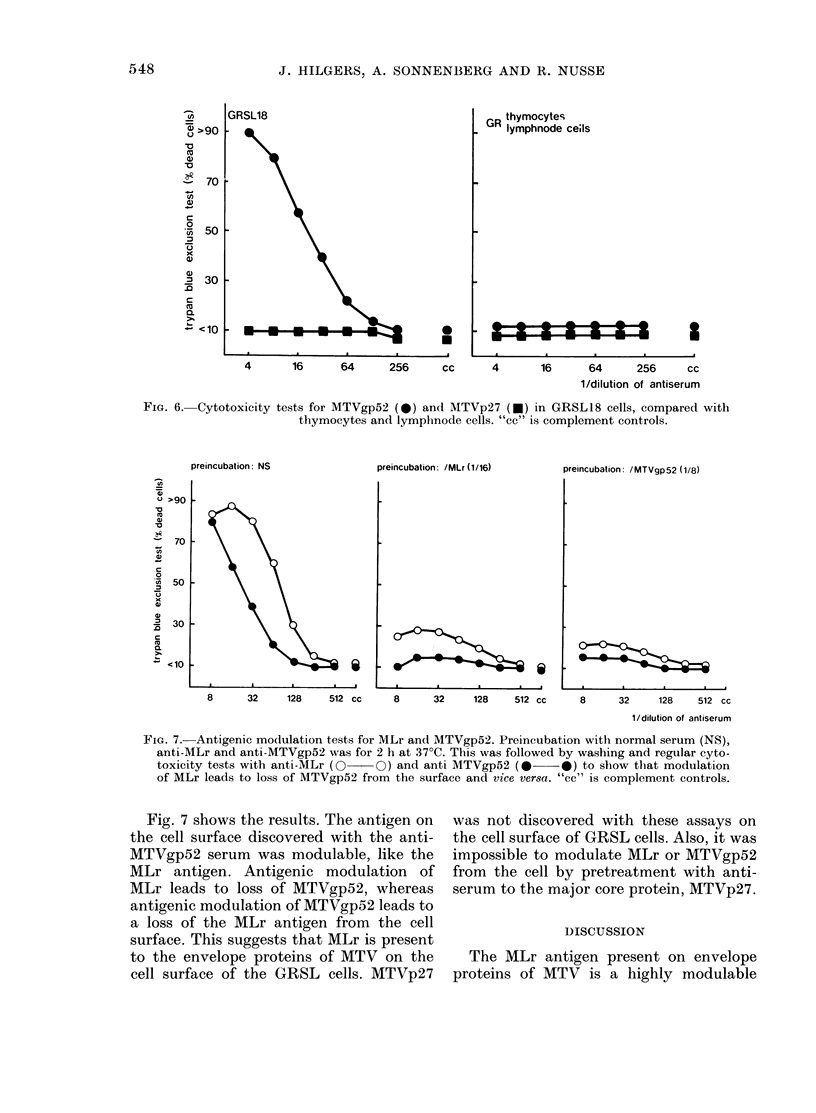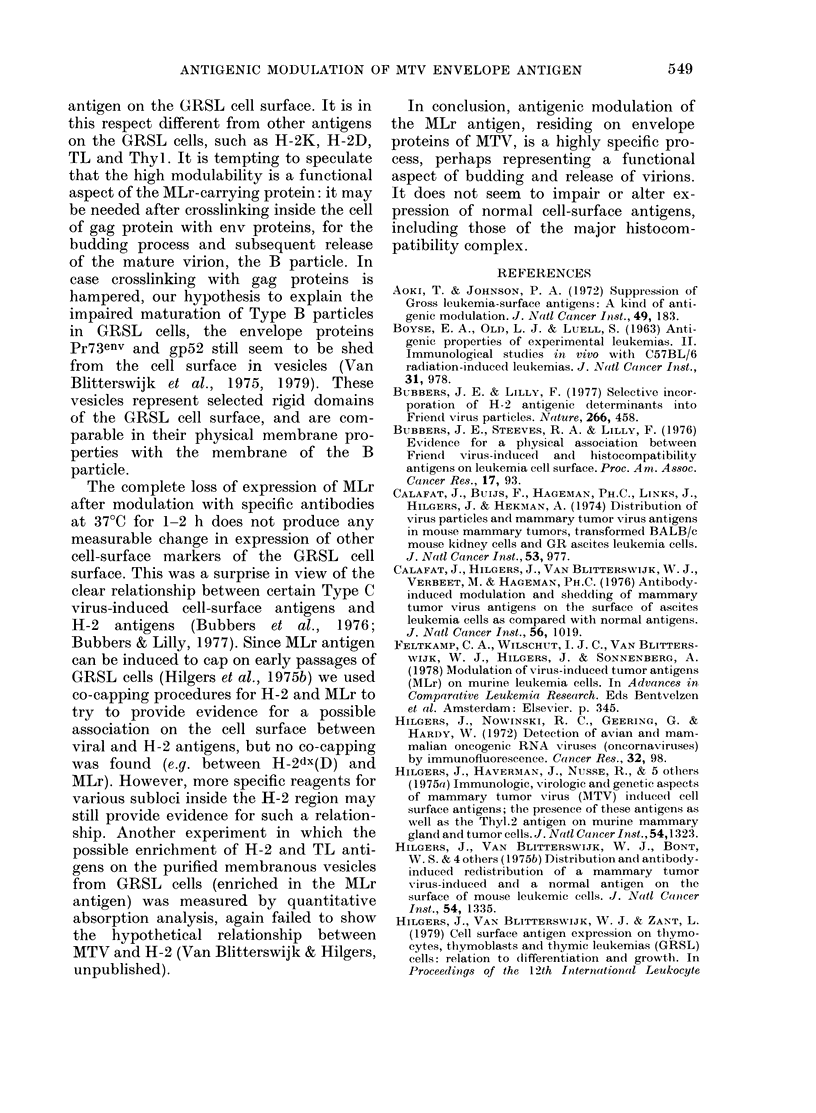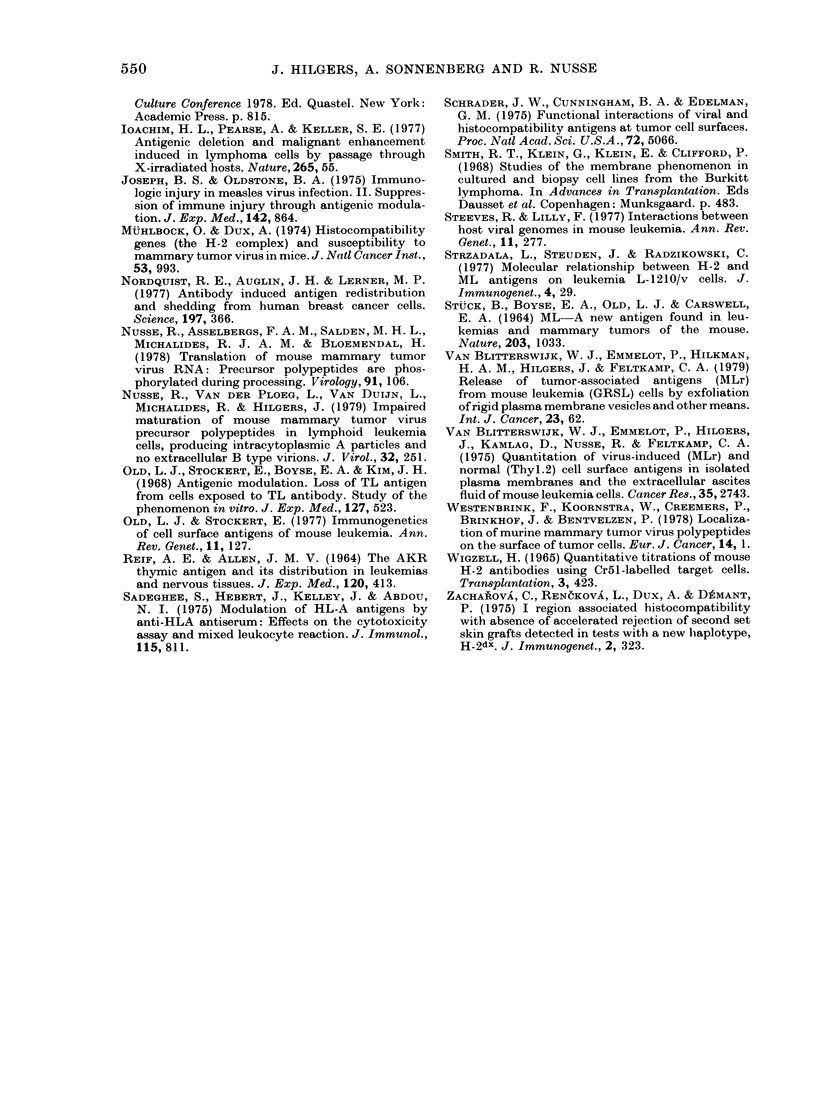# Antigenic modulation of mammary tumour virus envelope antigen or GR thymic lymphoma cells in relation to expressions of H-2, TL cell-surface antigens and THY1.

**DOI:** 10.1038/bjc.1980.278

**Published:** 1980-10

**Authors:** J. Hilgers, A. Sonnenberg, R. Nusse

## Abstract

The MLr antigen, a mammary tumour virus-induced antigen on the surface of GR thymic lymphoma cells (GRSL) can be modulated from the cell surface upon incubation with specific antiserum for 1-2 h at 37 degrees C, followed by washing the cells. In contrast, a number of other cell-surface antigens on these GRSL cells cannot be modulated under similar conditions. These antigens include histocompatibility antigens of the H-2 complex (H-2.8 of the K-end and H-2dx(D) of the H-2dx haplotype) and two thymic markers, TL1.2 and Thy1.2. Antigenic modulation of MLr as tested by trypan-blue exclusion and by chromium51 release does not lead to a measurable change in the expression of H-2K, H-2D, TL and Thy1.2 antigens. These results could be confirmed by absorption analysis. The latter analysis showed that the number of antigenic sites per cell are about the same for MLr and the two H-2 antigens, while TL antigens are scarcer and Thy1.2 antigens are more abundant. The procedure of antigenic modulation showed that the MLr antigen resides on MTVgp52, the major protein of the envelope. There was no evidence of internal proteins, such as MTVp27, on the surface of GRSL cells.


*Br. J. Cancer* (1980) **42**, 542

### ANTIGENIC MODULATION OF MAMMARY TUMOUR VIRUS ENVELOPE ANTIGEN ON GR THYMIC LYMPHOMA CELLS IN RELATION TO EXPRESSION OF H-2, TL CELL-SURFACE ANTIGENS AND THY1

J. HILGERS*, A. SONNENBERG AND R. NUSSE

*From the Divisions of Genetics and Virology, The Netherlands Cancer Institute, Antoni van Leeuwenhoekhuis, Plesmanlaan* 121, 1066 *CX Amsterdam, The Netherlands*

Received 14 March 1980 Accepted 27 June 1980

**Summary.—The MLr antigen, a mammary tumour virus-induced antigen on the surface of GR thymic lymphoma cells (GRSL) can be modulated from the cell surface upon incubation with specific antiserum for 1–2 h at 37°C, followed by washing the cells. In contrast, a number of other cell-surface antigens on these GRSL cells cannot be modulated under similar conditions. These antigens include histocompatibility antigens of the H-2 complex (H-2·8 of the K-end and H-2$^{dx}$(D) of the H-2$^{dx}$ haplotype) and two thymic markers, TL1·2 and Thy1·2. Antigenic modulation of MLr as tested by trypan-blue exclusion and by chromium$^{51}$ release does not lead to a measurable change in the expression of H-2K, H-2D, TL and Thy1·2 antigens. These results could be confirmed by absorption analysis. The latter analysis showed that the number of antigenic sites per cell are about the same for MLr and the two H-2 antigens, while TL antigens are scarcer and Thy1·2 antigens are more abundant.**

**The procedure of antigenic modulation showed that the MLr antigen resides on MTVgp52, the major protein of the envelope. There was no evidence of internal proteins, such as MTVp27, on the surface of GRSL cells.**

ANTIGENIC MODULATION of a cell-surface antigen is the phenomenon in which cells can become negative for the expression of an antigen after incubation at 37°C with a specific antibody. It was first described for the TL antigenic system in mice by Boyse *et al.* (1963) and confirmed by Old *et al.* (1968). It has since been reported for a number of apparently unrelated cell-surface antigens, such as Epstein–Barr virus-associated membrane antigens (Smith *et al.*, 1968), murine leukaemia virus-related antigens (Aoki & Johnson, 1972; Ioachim *et al.*, 1977), an antigen on human breast-cancer cells (Nordquist *et al.*, 1977), HL-A antigens on human leucocytes (Sadeghee *et al.*, 1975), measles virus-induced cell-surface antigen (Joseph & Oldstone, 1975) and, finally, for ML and MLr antigens related to murine mammary tumour virus by our group (Calafat *et al.*, 1976; Feltkamp *et al.*, 1978) and by Strzadala *et al.* (1977). For a recent review on the phenomenon of antigenic modulation, also called "lysostrip", especially for the TL antigens, see Old & Stockert (1977).

The question can be asked whether antigenic modulation of a cell-surface antigen leads to changes in expression of other antigens on the same cell surface. Would molecules adjacent to the modulated one show an increased expression, or would they "comodulate"? Since redistribution of antigens on crosslinking with bivalent antibodies precedes the modulation pro-

* Supported in part by Contract NO1-CP-33368 from the Division of Cancer Cause and Prevention, National Cancer Institute, U.S.A.

cess, it may be that surrounding antigens become redistributed too, a phenomenon comparable to co-capping (Schrader *et al.*, 1975). Old *et al.* (1968) were able to show that antigenic modulation of TL antigens leads to an increased expression of H-2D antigens, which are themselves non-modulable.

The "linkage" on the cell surface of viral and histocompatibility antigens may imply a mechanism of H-2-controlled susceptibility to the virus-induced neoplasm (for review, see Steeves & Lilly, 1977), as described in the murine Type C oncovirus system. Since the major histocompatibility complex (H-2D region) was shown to control resistance to mammary tumorigenesis by exogenous murine mammary-tumour viruses (Mühlbock & Dux, 1974) it was thought worth while to study the possible interaction, if it should occur, between the modulable mammary tumour virus-induced cell-surface antigens and the non- or hardly modulable antigens of the H-2K and H-2D end.

As an experimental model for such studies we chose ascites tumours in the GR mouse strain. These tumours are transplanted thymic lymphomas (GRSL cells) expressing the so-called MLr antigen (Hilgers *et al.*, 1975*a*) an antigen similar or even identical to ML present on thymic lymphomas of the DBA/2 mouse strain (Stück *et al.*, 1964; Strzadala *et al.*, 1977). Similar studies on the possible molecular relationship between mammary tumour virus-induced antigens and major histocompatibility antigens, were carried out on L-1210 cells from the DBA strain by Strzadala *et al.* (1977).

GRSL cells produce very few, if any, extracellular virions or Type B particles, but an abundance of intracytoplasmic A particles (Calafat *et al.*, 1974). The precursor polypeptide for MTVgag proteins (Pr73) does not seem to become phosphorylated in GRSL cells, and does not convert in the Pr76 peptide. The $Pr73^{gag}$ precursor protein is quite stable, is the major protein of the intracytoplasmic A particle and is not found on the cell surface. The precursor of the envelope proteins of MTV ($Pr73^{env}$) is also expressed in GRSL cells and present on the cell surface as shown by lactoperoxidase-catalysed labelling with $^{125}$iodine. This precursor is hardly processed, though some MTVgp52, the major envelope structural protein of MTV, can be found. These recent studies by our group (Nusse *et al.*, 1979) are an indication that the MLr antigen resides on proteins coded for by the env region of MTV. Similar conclusions can be drawn from the work of Westenbrink *et al.* (1978) for the ML antigen on L-1210 cells and for the MLr antigen on GRSL cells.

### MATERIALS AND METHODS

*Cells.*—Thymic lymphoma cells, called GRSL cells (Hilgers *et al.*, 1975*a*), were used as well as normal lymphoid cells of the GR strain. In the studies reported here the 18th spontaneous thymic lymphoma was used (GRSL18) between the 20–60th transplant generations in ascites. GRSL18 was maintained by weekly i.p. injections of $10^7$ cells, in young adult GR mice of either sex, washed ×3 in Earle's balanced salt solution after removal. The usual harvest of cells after one week of intraperitoneal growth was $3–9 \times 10^8$ cells.

*Antisera.*—All antisera were inactivated by incubation for 30 min at 56°C. The rabbit anti-MTV serum was prepared by 4 or more 1–3 monthly injections of purified, Tween-ether-treated B particles from C3H mammary tumours. The first injection was with incomplete Freund's adjuvant, and later injections with complete Freund's. The antiserum was absorbed *in vivo* by injecting 1 ml i.p. in an $MTV^-$ young male mouse, usually of the BALB/c strain, and recovered by bleeding the mouse the next day. It is thus a mixture of specific rabbit antibodies to MTV and mouse immunoglobulins. Normal rabbit serum was treated in the same way for control experiments. Such "rigorous" *in vivo* absorptions are needed to obtain specificity, and are superior to the usual *in vitro* absorption procedures as shown previously (Hilgers *et al.*, 1972).

The two antisera prepared against MTV

proteins purified according to described procedures (Nusse *et al.*, 1978) (*i.e.* anti-MTVgp52 and anti-MTVp27) were both absorbed *in vivo* before use in the cytotoxicity assays. They are both precipitating sera and exhibit strong immunofluorescence on fixed GRSL cells. Anti-MTVp27 shows a brilliant pattern resembling the distribution of intracytoplasmic A particles in the GRSL cells, while the anti MTVgp52 shows a more diffuse pattern on the fixed cells. Possible contaminating antibodies to other virion proteins are not detectable with conventional immunological assays like immunodiffusion and gelelectrophoresis following immune precipitation.

The antiserum to H-2·8 antigen was prepared by weekly injections of B10·M lymphoid cells in (B10 × A.SW)$F_1$ hybrid mice. H-2·8 represents a public specificity of the K-end in the H-$2^{dx}$ haplotype. An antiserum against the (largely unknown) D-end of the H-$2^{dx}$ halotype was prepared by injecting GR lymphoid cells in the (BALB/c × B10.M)$F_1$ hybrid. The antiserum is called H-$2^{dx}$(D). For details on the H-2 haplotype of the GR strain see Zachařová *et al.* (1975). The antiserum to the Thyl·2 antigen described first by Reif & Allen (1964) was prepared by weekly injections of thymocytes of the C3H/HeAf strain (Thyl·2) into the AKR/FuRdA (Thy-1·1) strain. Antiserum to TL antigens was prepared by weekly injections of A/BrA (TL1·2·3) thymocytes in A/$TL^-$/MA mice. Both A strains are of the H-$2^a$ haplotype. Analysis of the TL antigens present on GRSL cells and GR thymocytes is presented elsewhere (Hilgers *et al.*, 1979). GRSL cells show additional TL specificities, compared with thymocytes; they are most likely TL1·2·4, while thymocytes are TL2. Since the antiserum is against TL1·2·3 the TL antigens are designated TL1·2.

*Complement.*—Normal rabbit sera from selected rabbits were used as a source of complement. For absorption of naturally occurring heteroantibodies, 9 parts of complement were mixed with one part 0·1M EDTA. The mixture was incubated with mouse lymphoid cells for 1 h at 4°C on a shaker. After incubation the mixture was centrifuged and one part of 0·1M $CaCl_2$ was added before aliquotting and storage at −70°C. A second identical absorption using GRSL cells was occasionally necessary, to maintain the < 10% dead cells in the complement controls.

*Trypan-blue exclusion cytotoxicity test.*—Equal volumes of 20 μl cells ($5 \times 10^6$ cells/ml) antiserum dilutions and complement (1/3) were incubated for 45 min at 37°C in microtitre plates. After incubation the plates were put on ice and the number of dead cells counted immediately on addition of 20 μl 0·5% trypan blue. For absorption analysis, dilutions of cells (1/1 is $2 \times 10^8$ cells) with antiserum in the appropriate dilution at a ratio of 5:1 were incubated overnight at 4°C on a shaker. After incubation, the tubes were centrifuged for 15 min at 2400 *g* and 20 μl of supernatant was removed and put in microtitre wells for the trypan-blue test as well as the $^{51}$Cr-release assay.

*$^{51}$Cr-release test.*—One ml ($2 \times 10^7$) cells was labelled with 100–150 μCi Na-$^{51}$ chromate in phosphate-buffered saline. After 40 min incubation at 37°C in a waterbath on a shaker the cells were washed × 3 and resuspended till a concentration of $5 \times 10^6$ ml was reached. This was followed by a cytotoxicity test as described for the trypan-blue exclusion assay. After 45 min at 37°C the microtitre plates were put on ice and 140 μl medium was added to the 60 μl of reaction mixture present in each well. The amount of released isotype was counted for 4 min in a gammacounter and the results were expressed as the percentage of maximal isotope release, obtained by freezing and thawing × 3 the labelled GRSL cells.

The percentage of dead cells is now: released counts/total counts, where total counts is the count after freezing and thawing × 3 which, in fact, is about 88% of the total count in the cells. This method is described by Wigzell (1965).

*Antigenic modulation procedures.*—GRSL cells were washed × 3 with Earle's balanced salt solution. A total of $5 \times 10^6$ cells were preincubated at 37°C with the different sera. Aliquots of the cell were taken at 2, 5, 15, 30, 60 and 120 min and immediately cooled till 4°C on ice and diluted × 4 with Medium 199 containing 2% foetal calf serum. After centrifugation the aliquots were washed twice again before the cells were used in a trypan-blue exclusion or $^{51}$Cr-release assay. In other studies aliquots were taken at 120 min only and processed similarly. The preincubation was done under continuous shaking, and even extra shaking by hand with 15 min intervals. Shaking during preincubation and extensive washing thereafter are essential to obtain optimal results.

### RESULTS

Fig. 1 shows that preincubation with antiserum to MLr, followed by washing of the cells at different intervals, followed by incubation with normal serum (rabbit serum processed for *in vivo* absorption in the mouse) and complement in a regular cytotoxicity test (45 min at 37°C) leads to a rapid increase in killing of the target cells for about 15 min, and then a slow decrease in killing, extending over a period of 1–2 h. Two hours' preincubation with anti-MLr serum dilutions of 1/8 to 1/16 (titre of antiserum ranges from 1/32 to 1/128) is generally sufficient to "remove" all MLr from the cell surface, preventing the fixation of complement and subsequent death of the cells. Our previous studies (Van Blitterswijk *et al.*, 1975, 1979) have provided evidence that the MLr antigen is shed from the cell surface, rather than pinocytosed. Fig. 1 furthermore shows that preincubation with antiserum to MLr in a 1/8 dilution for 2 h, followed by washing and a cytotoxicity test with the same antiserum, leads to negativity for the antigen when compared to the control cells preincubated with normal serum.

FIG. 1.—Antigenic modulation of MLr antigen. GRSL18 cells were "preincubated" with antiserum to MLr (1/16), washed at various times after preincubation (a) and subsequently treated with complement in a regular trypan-blue exclusion test. "ns" refers to preincubations with normal serum for 120 min. (b) Preincubation with anti-MLr serum is followed by a cytotoxicity test with normal serum (○——○) and anti-MLr serum (●——●). "cc" is complement controls.

From Fig. 2 it appears that antigenic modulation cannot be detected under comparable circumstances for all other antigens tested; H-2K and H-2D antigens of the H-$2^{dx}$ haplotype are non-modulable on GRSL cells, and the same holds true for the thymic markers TL1·2 and Thy1·2. It may seem surprising that no modula-

FIG. 2.—Antigenic modulation tests for various antigens. Preincubation was carried out with antisera against MLr, H-2·8(K), H-$2^{dx}$ (D), Thy1·2 and TL1·2·3 with, for each antigen, normal sera as control (○——○ or □——□). In all cases except MLr, the preincubation with antiserum followed by incubation with the same antiserum leads to higher cytotoxicity than in the control situation. "cc" is complement controls.

FIG. 3.—Specificity of antigenic modulation of MLr. Preincubation with anti-MLr (●) serum and control serum (○) for 120 min is followed by cytotoxicity tests for MLr, H-2·8(K), TL1·2 and Thy1·2. Two types of cytotoxicity tests (trypan blue and $^{51}$Cr-release) were applied on the same batch of preincubated GRSL18 cells, as described in Materials and Methods. "cc" is complement controls.

FIG. 4.—Specificity of antigenic modulation of MLr compared with H-2$^{dx}$(D) antigen. Preincubation with anti-MLr, anti H-2$^{dx}$ (D) and normal serum (NS) for 120 min was followed by washing and cytotoxicity tests with anti-MLr (●——●) and anti-H-2$^{dx}$(D) (○——○). "cc" is complement controls.

FIG. 5.—Specificity of antigenic modulation of MLr, measured by cytotoxic absorption. Preincubation with anti-MLr (●——●) and normal serum (○——○) was for 2 h at 37°C. Counted numbers of cells were then used for absorption analysis (target cells for MLr, H-2·8(K) and TL1.2 were GRSL18 cells, whilst thymocytes were used as target cells for Thyl·2). Note that the number of cells for 50% absorption for the various antigens is $\pm 4 \times 10^7$ (MLr), $\pm 4 \times 10^7$ (H-2·8), $\pm 16 \times 10^7$ (TL1·2), $\pm 4 \times 10^6$ (Thyl·2). This implies that the density of Thyl·2 antigenic sites is much higher than that of MLr and H-2·8 (both $\times$ 10 less) and TL1·2 ($\times$ 40 less). "cc" is complement controls.

tion was found for TL antigens on these leukaemia cells, the antigenic system with which this phenomenon was first discovered. However, it has been reported that TL antigens are non-modulable on a variety of target cells, whilst they are modulable on other cell types.

Since it has recently been observed that certain interactions between cell-surface antigens occur on the cell surface of a variety of lymphoid cells, notably between viral and histocompatibility antigens, it seemed worth while to study whether antigenic modulation of the viral antigen, MLr, would lead to a change in expression of histocompatibility or other antigens. Such experiments are presented in Fig. 3, using both the conventional trypan-blue exclusion test and a $^{51}$Cr-release assay. It can be seen that both assays record the antigenic modulation process, although this is somewhat less clear for the $^{51}$Cr-release assay.

If MLr modulated cells are tested for the expression of H-2K, TL1·2 and Thyl·2, as shown in Fig. 3 by the 2 tests, no significant change can be found from the control cell preincubated with normal serum. Fig. 4 shows this for H-2$^{dx}$(D) and also shows again that H-2D antigens are non-modulable on GRSL cells. These results could be confirmed by absorption analysis, as shown in Fig. 5. No changes in absorption capacity of modulated cells compared to control cells could be observed for H-2K, TL1·2 and Thyl·2. Note in this figure that the number of antigenic sites varies considerably (though it is hard to determine exact numbers by absorption analysis, and these numbers depend on the exact dilution of antiserum used for absorption). MLr appears to be present on the cell surface in comparable density to H-2 antigens, less dense than Thyl·2 antigen and denser than TL1·2 antigen. Comparable densities are found for H-2K and H-2D antigens (results not shown).

It was still doubtful whether the MLr antigen resides on envelope proteins of MTV (MTVgp52) only. Recent advances in the preparation of antisera against purified proteins of MTV opened the possibility of studying this question. It appears that rabbit anti-MTVgp52 is clearly cytotoxic for GRSL, whilst anti-MTVp27 is not (Fig. 6). The anti-MTVgp52 serum does not react with normal thymocytes or lymphnode cells, suggesting MTV specificity, at least after *in vivo* absorption. Since gp52 is known to be an envelope protein and p27 a core protein of MTV, it seemed likely that the MLr antigen would reside on an envelope protein or its precursor. The phenomenon of antigenic modulation found for the MLr antigen was used to test this hypothesis further.

FIG. 6.—Cytotoxicity tests for MTVgp52 (●) and MTVp27 (■) in GRSL18 cells, compared with thymocytes and lymphnode cells. "cc" is complement controls.

FIG. 7.—Antigenic modulation tests for MLr and MTVgp52. Preincubation with normal serum (NS), anti-MLr and anti-MTVgp52 was for 2 h at 37°C. This was followed by washing and regular cytotoxicity tests with anti-MLr (○——○) and anti MTVgp52 (●——●) to show that modulation of MLr leads to loss of MTVgp52 from the surface and *vice versa*. "cc" is complement controls.

Fig. 7 shows the results. The antigen on the cell surface discovered with the anti-MTVgp52 serum was modulable, like the MLr antigen. Antigenic modulation of MLr leads to loss of MTVgp52, whereas antigenic modulation of MTVgp52 leads to a loss of the MLr antigen from the cell surface. This suggests that MLr is present to the envelope proteins of MTV on the cell surface of the GRSL cells. MTVp27 was not discovered with these assays on the cell surface of GRSL cells. Also, it was impossible to modulate MLr or MTVgp52 from the cell by pretreatment with anti-serum to the major core protein, MTVp27.

### DISCUSSION

The MLr antigen present on envelope proteins of MTV is a highly modulable antigen on the GRSL cell surface. It is in this respect different from other antigens on the GRSL cells, such as H-2K, H-2D, TL and Thy1. It is tempting to speculate that the high modulability is a functional aspect of the MLr-carrying protein: it may be needed after crosslinking inside the cell of gag protein with env proteins, for the budding process and subsequent release of the mature virion, the B particle. In case crosslinking with gag proteins is hampered, our hypothesis to explain the impaired maturation of Type B particles in GRSL cells, the envelope proteins $Pr73^{env}$ and gp52 still seem to be shed from the cell surface in vesicles (Van Blitterswijk *et al.*, 1975, 1979). These vesicles represent selected rigid domains of the GRSL cell surface, and are comparable in their physical membrane properties with the membrane of the B particle.

The complete loss of expression of MLr after modulation with specific antibodies at 37°C for 1–2 h does not produce any measurable change in expression of other cell-surface markers of the GRSL cell surface. This was a surprise in view of the clear relationship between certain Type C virus-induced cell-surface antigens and H-2 antigens (Bubbers *et al.*, 1976; Bubbers & Lilly, 1977). Since MLr antigen can be induced to cap on early passages of GRSL cells (Hilgers *et al.*, 1975*b*) we used co-capping procedures for H-2 and MLr to try to provide evidence for a possible association on the cell surface between viral and H-2 antigens, but no co-capping was found (*e.g.* between $H\text{-}2^{dx}$(D) and MLr). However, more specific reagents for various subloci inside the H-2 region may still provide evidence for such a relationship. Another experiment in which the possible enrichment of H-2 and TL antigens on the purified membranous vesicles from GRSL cells (enriched in the MLr antigen) was measured by quantitative absorption analysis, again failed to show the hypothetical relationship between MTV and H-2 (Van Blitterswijk & Hilgers, unpublished).

In conclusion, antigenic modulation of the MLr antigen, residing on envelope proteins of MTV, is a highly specific process, perhaps representing a functional aspect of budding and release of virions. It does not seem to impair or alter expression of normal cell-surface antigens, including those of the major histocompatibility complex.

REFERENCES

AOKI, T. & JOHNSON, P. A. (1972) Suppression of Gross leukemia-surface antigens: A kind of antigenic modulation. *J. Natl Cancer Inst.*, **49**, 183.

BOYSE, E. A., OLD, L. J. & LUELL, S. (1963) Antigenic properties of experimental leukemias. II. Immunological studies *in vivo* with C57BL/6 radiation-induced leukemias. *J. Natl Cancer Inst.*, **31**, 978.

BUBBERS, J. E. & LILLY, F. (1977) Selective incorporation of H-2 antigenic determinants into Friend virus particles. *Nature*, **266**, 458.

BUBBERS, J. E., STEEVES, R. A. & LILLY, F. (1976) Evidence for a physical association between Friend virus-induced and histocompatibility antigens on leukemia cell surface. *Proc. Am. Assoc. Cancer Res.*, **17**, 93.

CALAFAT, J., BUIJS, F., HAGEMAN, PH.C., LINKS, J., HILGERS, J. & HEKMAN, A. (1974) Distribution of virus particles and mammary tumor virus antigens in mouse mammary tumors, transformed BALB/c mouse kidney cells and GR ascites leukemia cells. *J. Natl Cancer Inst.*, **53**, 977.

CALAFAT, J., HILGERS, J., VAN BLITTERSWIJK, W. J., VERBEET, M. & HAGEMAN, PH.C. (1976) Antibody-induced modulation and shedding of mammary tumor virus antigens on the surface of ascites leukemia cells as compared with normal antigens. *J. Natl Cancer Inst.*, **56**, 1019.

FELTKAMP, C. A., WILSCHUT, I. J. C., VAN BLITTERSWIJK, W. J., HILGERS, J. & SONNENBERG, A. (1978) Modulation of virus-induced tumor antigens (MLr) on murine leukemia cells. In *Advances in Comparative Leukemia Research.* Eds Bentvelzen *et al.* Amsterdam: Elsevier. p. 345.

HILGERS, J., NOWINSKI, R. C., GEERING, G. & HARDY, W. (1972) Detection of avian and mammalian oncogenic RNA viruses (oncornaviruses) by immunofluorescence. *Cancer Res.*, **32**, 98.

HILGERS, J., HAVERMAN, J., NUSSE, R., & 5 others (1975*a*) Immunologic, virologic and genetic aspects of mammary tumor virus (MTV) induced cell surface antigens; the presence of these antigens as well as the Thy1.2 antigen on murine mammary gland and tumor cells. *J. Natl Cancer Inst.*, **54**, 1323.

HILGERS, J., VAN BLITTERSWIJK, W. J., BONT, W. S. & 4 others (1975*b*) Distribution and antibody-induced redistribution of a mammary tumor virus-induced and a normal antigen on the surface of mouse leukemic cells. *J. Natl Cancer Inst.*, **54**, 1335.

HILGERS, J., VAN BLITTERSWIJK, W. J. & ZANT, L. (1979) Cell surface antigen expression on thymocytes, thymoblasts and thymic leukemias (GRSL) cells: relation to differentiation and growth. In *Proceedings of the 12th International Leukocyte*

*Culture Conference* 1978. Ed. Quastel. New York: Academic Press. p. 815.

IOACHIM, H. L., PEARSE, A. & KELLER, S. E. (1977) Antigenic deletion and malignant enhancement induced in lymphoma cells by passage through X-irradiated hosts. *Nature*, **265**, 55.

JOSEPH, B. S. & OLDSTONE, B. A. (1975) Immunologic injury in measles virus infection. II. Suppression of immune injury through antigenic modulation. *J. Exp. Med.*, **142**, 864.

MÜHLBOCK, O. & DUX, A. (1974) Histocompatibility genes (the H-2 complex) and susceptibility to mammary tumor virus in mice. *J. Natl Cancer Inst.*, **53**, 993.

NORDQUIST, R. E., AUGLIN, J. H. & LERNER, M. P. (1977) Antibody induced antigen redistribution and shedding from human breast cancer cells. *Science*, **197**, 366.

NUSSE, R., ASSELBERGS, F. A. M., SALDEN, M. H. L., MICHALIDES, R. J. A. M. & BLOEMENDAL, H. (1978) Translation of mouse mammary tumor virus RNA: Precursor polypeptides are phosphorylated during processing. *Virology*, **91**, 106.

NUSSE, R., VAN DER PLOEG, L., VAN DUIJN, L., MICHALIDES, R. & HILGERS, J. (1979) Impaired maturation of mouse mammary tumor virus precursor polypeptides in lymphoid leukemia cells, producing intracytoplasmic A particles and no extracellular B type virions. *J. Virol.*, **32**, 251.

OLD, L. J., STOCKERT, E., BOYSE, E. A. & KIM, J. H. (1968) Antigenic modulation. Loss of TL antigen from cells exposed to TL antibody. Study of the phenomenon *in vitro*. *J. Exp. Med.*, **127**, 523.

OLD, L. J. & STOCKERT, E. (1977) Immunogenetics of cell surface antigens of mouse leukemia. *Ann. Rev. Genet.*, **11**, 127.

REIF, A. E. & ALLEN, J. M. V. (1964) The AKR thymic antigen and its distribution in leukemias and nervous tissues. *J. Exp. Med.*, **120**, 413.

SADEGHEE, S., HEBERT, J., KELLEY, J. & ABDOU, N. I. (1975) Modulation of HL-A antigens by anti-HLA antiserum: Effects on the cytotoxicity assay and mixed leukocyte reaction. *J. Immunol.*, **115**, 811.

SCHRADER, J. W., CUNNINGHAM, B. A. & EDELMAN, G. M. (1975) Functional interactions of viral and histocompatibility antigens at tumor cell surfaces. *Proc. Natl Acad. Sci. U.S.A.*, **72**, 5066.

SMITH, R. T., KLEIN, G., KLEIN, E. & CLIFFORD, P. (1968) Studies of the membrane phenomenon in cultured and biopsy cell lines from the Burkitt lymphoma. In *Advances in Transplantation*. Eds Dausset *et al.* Copenhagen: Munksgaard. p. 483.

STEEVES, R. & LILLY, F. (1977) Interactions between host viral genomes in mouse leukemia. *Ann. Rev. Genet.*, **11**, 277.

STRZADALA, L., STEUDEN, J. & RADZIKOWSKI, C. (1977) Molecular relationship between H-2 and ML antigens on leukemia L-1210/v cells. *J. Immunogenet.*, **4**, 29.

STÜCK, B., BOYSE, E. A., OLD, L. J. & CARSWELL, E. A. (1964) ML—A new antigen found in leukemias and mammary tumors of the mouse. *Nature*, **203**, 1033.

VAN BLITTERSWIJK, W. J., EMMELOT, P., HILKMAN, H. A. M., HILGERS, J. & FELTKAMP, C. A. (1979) Release of tumor-associated antigens (MLr) from mouse leukemia (GRSL) cells by exfoliation of rigid plasma membrane vesicles and other means. *Int. J. Cancer*, **23**, 62.

VAN BLITTERSWIJK, W. J., EMMELOT, P., HILGERS, J., KAMLAG, D., NUSSE, R. & FELTKAMP, C. A. (1975) Quantitation of virus-induced (MLr) and normal (Thy1.2) cell surface antigens in isolated plasma membranes and the extracellular ascites fluid of mouse leukemia cells. *Cancer Res.*, **35**, 2743.

WESTENBRINK, F., KOORNSTRA, W., CREEMERS, P., BRINKHOF, J. & BENTVELZEN, P. (1978) Localization of murine mammary tumor virus polypeptides on the surface of tumor cells. *Eur. J. Cancer*, **14**, 1.

WIGZELL, H. (1965) Quantitative titrations of mouse H-2 antibodies using Cr51-labelled target cells. *Transplantation*, **3**, 423.

ZACHAŘOVÁ, C., RENČKOVÁ, L., DUX, A. & DÉMANT, P. (1975) I region associated histocompatibility with absence of accelerated rejection of second set skin grafts detected in tests with a new haplotype, H-$2^{dx}$. *J. Immunogenet.*, **2**, 323.